# A meta-analysis of salivary cortisol responses in the Trier Social Stress Test to evaluate the effects of speech topics, sex, and sample size

**DOI:** 10.1016/j.cpnec.2022.100125

**Published:** 2022-02-10

**Authors:** Haixia Gu, Xue'er Ma, Jingjing Zhao, Chunyu Liu

**Affiliations:** a School of Psychology, Shaanxi Normal University, Xi'an, 710062, China; b Department of Psychiatry, SUNY Upstate Medical University, Syracuse, NY, United States

**Keywords:** Stress, Cortisol, Trier social stress test (TSST), Sample size, Meta-analysis

## Abstract

**Background:**

The Trier Social Stress Test (TSST) is one of the most widely used laboratory-based psychological stress paradigms. Previous studies have shown that males have a more robust cortisol response than females in the TSST. However, the effects of sample size, speech topic, and interaction between sex and speech topic on cortisol responses in TSST remain elusive. Our goal was to evaluate these influencing factors in the TSST using salivary cortisol reactivity as an objective measure.

**Methods:**

We collected TSST research articles in Web of Science, PubMed, PsycNet, and CNKI. We only included TSST studies that had measures of salivary cortisol both before and after task completion. A total of 65 articles involving 76 sub-studies met our inclusion criteria, with a total of 5171 participants (2040 females and 3131 males). The effects of sample sizes were assessed to determine if results of studies with various sample sizes were stable. We performed multivariate meta-regression to determine the effects of speech topic, sex, and the interaction between sex and speech topic after controlling their confounding effects. Subgroup analysis of sex was conducted to detect inter-group differences. We further evaluated the baseline and peak salivary cortisol concentrations for males and females independently to detect the sources of sex differences.

**Results:**

The average effect size (i.e., Cohen's *d*) of salivary cortisol reactivity was 0.93, 95% CI = 0.82 to 1.04, *p* < .001. The small studies produced larger variations in the reported effect sizes than the large-sample studies (*r* = -0.24, *p* = .041). A sample size of 40 was necessary to provide sufficient statistical power to detect significant changes of salivary cortisol in TSST. Speech topics, sex, and sex-speech topic interaction could predict salivary cortisol responses (F(*df1* = 3, *df2* = 72) = 11.98, *p* < .001) and explained 42.68% of the total experimental variation. Sex was the only significant contributing factor (*p* < .00025) in the regression model. Salivary cortisol responses in males were significantly higher than in females (*Q*_*B*_ = 42.89, *df* = 1, *p* < .001). Further, significant differences between males and females were detected at baseline (*t* = -2.03, *df* = 74, *p* = .046) and peak (*t* = -4.96, *df* = 74, *p* < .001).

**Conclusions:**

The TSST effectively induces stress response as measured by salivary cortisol change. Forty samples is the minimum sample size for detecting the robust salivary cortisol responses. We confirmed that males have more robust salivary cortisol reactivity than females in TSST. Speech topics that we tested did not significantly contribute to differences in salivary cortisol responses. No significant interaction between sex and speech topic on salivary cortisol responses was detected.

## Highlights

• Forty samples is the minimum for detecting robust salivary cortisol responses.
• The speech topics tested did not significantly alter salivary cortisol responses.
• Males have higher salivary cortisol responses than females in the TSST.
• Males had higher salivary cortisol levels than females in the TSST at both baseline and peak.

## 1 Introduction

All people face various forms of psychological stress in daily life. Individual stress response helps predict health and well-being [1]. A reliable and efficient acute stress testing paradigm is necessary to understand the mechanisms of physiological, psychological, neurobiological, and molecular responses to acute stress.

The Trier Social Stress Test (TSST) is recognized as the gold standard for inducing acute laboratory stress [2]. Kirschbaum et al. [3] first proposed the TSST, which is based on a mock public speaking task and a mental arithmetic task in front of an audience. The TSST as originally proposed includes a 30-min rest, a 10-min task preparation, a 5-min public speaking task, a 5-min mental arithmetic task, and 30–70 min for recovery. TSST characterised by two key important factors (i.e., social-evaluative threat and uncontrollability) could effectively elicit hypothalamic-pituitary-adrenal (HPA) axis stress responses [4,5]. Although cortisol levels may be regulated by many factors, in general cortisol is one of the most reliable biomarkers of the HPA axis [2,[6], [7], [8], [9]].

Since the introduction of the TSST, researchers have been trying to improve its performance and gain a deeper understanding of factors that impact its results. Previous studies have discussed or quantified individual factors (e.g., sex, age, Body Mass Index (BMI), menstrual cycle phase, and the use of oral contraceptive), and protocol parameters of TSST (e.g., time of day, acclimation duration, the difficulty of mental arithmetic, jury's size and feedback) for their effects on stress induction [2,[10], [11], [12], [13], [14]]. Because the methodology and other major variations of TSST have been reviewed previously [12,13], we focused on evaluating the robustness of TSST and parameters that have been commonly reported but not been assessed for their contribution to cortisol response, including speech topics and sample sizes. Sex is a known dominant influencing factor and was included in this trial to evaluate its interaction with speech topics [10].

Salivary cortisol is the preferred stress indicator in the TSST because it is simple to collect, non-invasive, and objective, as opposed to serum cortisol, which requires intravenous catheter placement that may affect stress responses [4,15]. Salivary cortisol, reflecting the levels of biologically active, non-protein bound cortisol in serum [9], is present in less than one-tenth of the concentration found in serum [16]. Some previous meta-analyses of TSST mixed data of salivary and plasma cortisol, which increased heterogeneity among studies and made the comparisons difficult [11,17]. Thus, we focused on using salivary cortisol as the evaluation index to quantify the acute stress responses induced by TSST in the current meta-analysis.

### 1.1 Speech topics

Speech topic is the core component of the TSST public speaking task. Several different topics have been tested. The job interview topic as described when TSST was introduced, is still the most commonly used topic [11,13]. Alternative speech topics that have since been tested include defending against an accusation of shoplifting or traffic violation [[18], [19], [20], [21], [22], [23], [24], [25], [26], [27], [28], [29]], introducing one's self to new classmates [30,31], imagining experiencing unfair treatment due to personal attributes [32], and describing a generic event or personally relevant event [33]. Some researchers attempt to modify the speech topics of the TSST protocol to enhance the stress response [19,23]. Although not all modifications for speech topics are designed to enhance the stress response, it is necessary to look for convincing evidence to explain the effects of speech topics in TSST studies.

Comparative studies on cortisol reactivity between speech topics have been completed with inconsistent and even contradictory findings [19,20,22,33,34]. Goodman et al. [11] and Linares et al. [13] claimed job interview is the most appropriate speech topic for robust stress responses. Still, they did not quantitatively evaluate the effects of different speech topics. The meta-analysis of TSST studies by Liu et al. [14] used four studies of modified speech topics. But three of the four studies were from the same study with only 84 independent participants. The sample size is too small to draw a solid conclusion about the effect size of the modified speech topics. Now, we compiled 358 independent samples to re-evaluate the effects of speech topics.

### 1.2 Sex

TSST protocol induces robust HPA axis responses in a wide range of age groups in both sexes [35]. In general, males have a stronger cortisol response than females in the TSST [14,36]. Compared with males of the same age, females between puberty and menopause have lower HPA axis responses [37]. Males' cortisol responses have been reported to be 1.5 to 2 times higher than age-matched females' when employing the public speaking and mental arithmetic tasks [38]. Cross and Madson [39] described the sex differences of self-construals, that males have independent self-construal, but females have interdependent self-construal. Males' and females' different self-construals are responsible for the sex differences of motivation, emotion, cognition, and social behavior. It was demonstrated that sex has a potential interaction with the nature of tasks. Males showed higher salivary cortisol reactivity to the public speaking and mental arithmetic tasks than females, which were referred to as achievement challenges and involved more “fight or flight” responses [40]. Thus, participants’ biological sex should be considered as a crucial covariate in TSST studies.

Whether males and females perform differently with the distinct speech topic in TSST is a research gap worthy of investigation. Males and females may show different sensitivities to the same speech topic, as suggested by a previous study of sex-related differences of stress-response to the same stress task [40]. Such interaction between sex and speech topics exists could potentially confound the study and increase the heterogeneity of TSST.

### 1.3 Sample sizes

The sample sizes varied from a dozen to hundreds in TSST studies. Coupled with publication bias, there is an upward bias known as the “winner's curse”, where the effect size can be overestimated in the small studies. Subsequent studies cannot reproduce significant findings from the initial studies [[41], [42], [43]]. At the same time, small under-powered studies can fail to detect the effect. A meta-analysis accumulates data from multiple studies to maximize power and stabilize the findings. Although TSST has been used to elicit acute stress responses for decades, it is not clear how big a study is needed to produce stable results of salivary cortisol response. With the data collected, we have an opportunity to investigate if the effect size is stable over various sample sizes; what the true effect size is and the minimum sample size required for reproducible results. The answers can help researchers decide sample size for future studies and appropriately interpret the results.

In this meta-analysis, we evaluated the effects of sample sizes and tested whether and how speech topics, sex, and their interaction affect the salivary cortisol responses with a large collection of TSST studies. The results were expected to clarify how these factors contribute to the results of TSST and ultimately improve reproducibility of the test.

## 2 Methods

### 2.1 Literature search strategy

This meta-analysis included TSST studies on healthy adults with salivary cortisol measures. Online searches were conducted on April 21, 2020 using PubMed, PsycNet, Web of Science, and China National Knowledge Infrastructure (CNKI) by the first author (Gu. H.). All English and Chinese articles were collected from November 1993 (first publication on TSST) to April 2020. MeSH terms, i.e., “Trier Social Stress Test”, “Cortisol”, and their Entry terms were the search words used (**Appendix A. Search strategies**).

### 2.2 Inclusion/exclusion criteria

Research articles had to meet four criteria for inclusion in our study: a) participants were healthy adults; b) the TSST protocol was used. The TSST protocol should have both the public speaking and mental arithmetic tasks and introduce the on-site audience who provide neutral or negative nonverbal feedback; c) participants were tested in the afternoon; d) salivary cortisol samples were collected before and after the public speaking and mental arithmetic tasks.

Many factors caused article exclusion including a) reviews, conference reports, academic dissertations, or overlapping data (when overlapping data was discovered, the article providing larger sample size and clearest information was kept); b) participants experienced additional stressors or other interventions before and during TSST (e.g., cognitive tests, physical activity, eating, Cold Pressor Task (CPT)); c) participants’ mean age was less than 18 or greater than 65; d) the TSST was performed in the morning or evening. We restricted the choice of studies to those completed between lunch and dinner because cortisol is markedly modulated by circadian rhythm and eating; e) the paradigm was excessively modified from the original protocol proposed by Kirschbaum et al. [3] as missing one of the three core TSST components. Minor modifications are allowed in, such as duration of certain periods (the studies without anticipation and recovery periods were included), the difficulty of tasks, jury size, and the number of participants in each experiment; f) no salivary cortisol data reported by sex grouping.

**Fig. 1:**
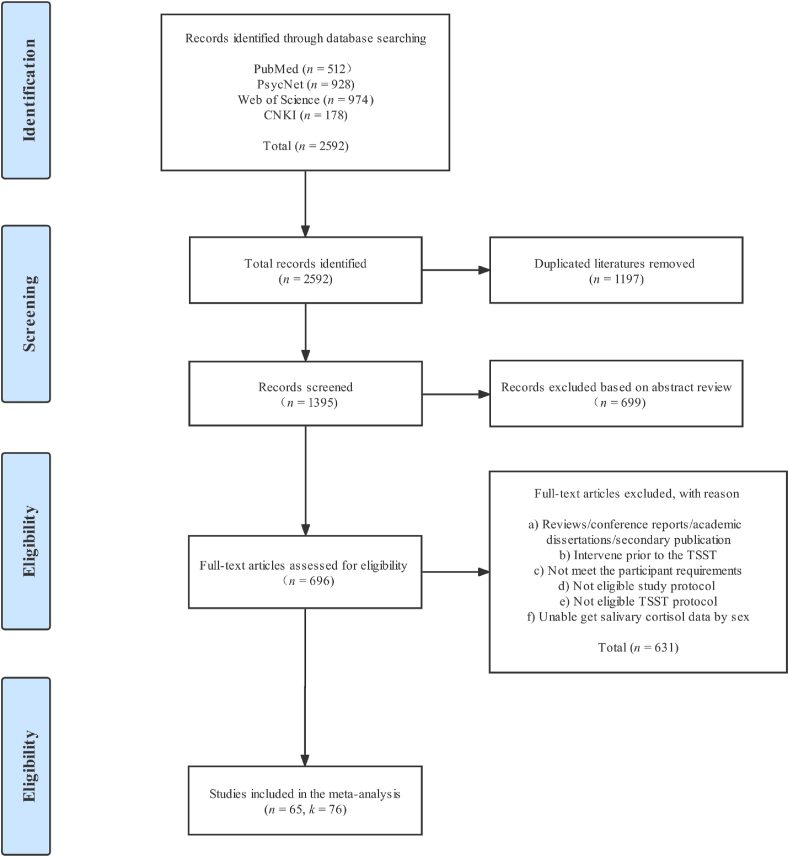
**Preferred reporting items for meta-analysis (PRISMA) flowchart.***n* represents the number of articles, and *k* represents the number of sub-studies.

The literature search identified 2592 articles for consideration. Of these, 1197 studies were removed for duplication. The remaining 1395 articles were further screened by the above inclusion and exclusion criteria, with 696 articles removed after abstract screening, and 631 articles removed after full-text screening, leaving 65 articles for further meta-analysis (Fig. 1). The first author (Gu. H.) reviewed all the articles; the second author (Ma. X.) randomly independently reviewed 15% of articles. We then compared coding and resolved disputes. Further, we examined the reference lists of meta-analyses of TSST studies, and no articles were added for the final meta-analysis. EndNote X9 software was used for article management.

Two technicians (Gu. H. & Ma. X.) independently implemented the quality assessment for the 65 selected articles according to the 14-item Checklist for assessing the quality of quantitative studies (QualSyst) [44]. Eleven of the fourteen criteria were applied to all articles, and the randomization criterion was used when applicable. Two of the fourteen criteria, blinding of investigators and blinding of subjects, were not used. The mean scores of quality assessment were 92.21%, with a range of 70.83%–100%. No article was excluded for poor quality, with a quality score of all articles greater than 55%. When technicians differed in article assessments, conflicts were discussed until agreements were reached, and the details were provided in Appendix B**. Quality assessment**.

### 2.3 Data extraction

One technician (Gu. H.) coded all 76 sub-studies across 65 articles on speech topics, sex, sample sizes, mean age, the mean (*M*) and standard deviation (*SD*) of salivary cortisol at baseline and peak. The baseline is the minimum of salivary cortisol that the researchers can measure before introducing the speech task, which usually was taken after the acclimation period [11,14]. The peak is the highest salivary cortisol value that the researchers can measure after completing both tasks, usually was taken during the recovery period [4,14]. These differences of the two samples (baseline and peak salivary cortisol) reflect the salivary cortisol response in the TSST protocol. Another technician (Ma. X.) independently randomly coded 50 sub-studies from 40 articles. The technicians then compared coding and resolved disputes. The Pearson's correlation on cortisol data between the two technicians was 0.970, *p* < .001. The major variables were speech topics (job interview and other speech topics), sex (male and female), and sample sizes of the job interview group. The number of studies that met inclusion criteria with modified speech topics was small, including defending against an accusation of shoplifting (*k* = 2), introducing one's self to new classmates (*k* = 2), imagining experiencing unfair treatment due to personal attributes (*k* = 1). Therefore, we combined data of other speech topics into one group to compare to data of job interview.

Baseline and peak salivary cortisol data were extracted for calculating the cortisol responses. When articles did not provide numerical salivary cortisol data, we requested the data from the manuscript authors. When we failed to get a response from the original authors, WebPlotDigitizer [45] was used to extract the *M* and *SD* from the figures in the publications. We acquired the raw data of 14 articles (21.54%) from the publications and 10 articles (15.38%) from the manuscript authors. The data of the remaining 41 articles (63.08%) were extracted from the figures. *SD* was calculated from the standard error (*SE*) following the formula: *SD* = *SE* * $\sqrt{N}$, where *N* represents the sample size. The units of salivary cortisol concentration were converted to nmol/l (nanomoles per liter). We merged the raw salivary cortisol data for variables not of interest in this study (e.g., sleep quality, genotypes) following the formula: $M=\frac{\sum_{i=1}^{m}N_{i}M_{i}}{\sum_{i=1}^{m}N_{i}}$, $SD=\sqrt{\frac{\sum_{i=1}^{m}\left(N_{i}\;-\;1\right)S{D^{2}}_{i}\;+\;\sum_{i=1}^{m}N_{i}{\left(M_{i}\;-\;M\right)}^{2}}{\sum_{i=1}^{m}N_{i}\;-\;1}}$ (Zhang et al., 2016). Ultimately, 65 articles involving 76 sub-studies were used for further analysis.

### 2.4 Data analysis

To identify the effects of speech topics and sex, multivariate meta-regression and subgroup analysis were used. Multivariate meta-regression was conducted initially to determine the impact of speech topics, sex, and the interaction between speech topics and sex on salivary cortisol response. The regression model was represented by the equation: Salivary cortisol ∼ speech topics + sex+ speech topics * sex + intercept. The restricted maximum likelihood (REML) method was used to estimate the between-study variance (Patterso and Thompson, 1971). Further, subgroup analysis was completed to identify inter-subgroup differences (*Q*_*B*_) and the sources of study heterogeneity (*I*^*2*^). Additionally, to demonstrate the source of sex differences, we independently evaluated the female-male differences in salivary cortisol concentration at baseline and peak by *t*-test.

To determine the influence of sample size, we checked its effects on salivary cortisol responses in studies using the job interview topic. The effect size variation in each study was calculated: effect size variation = mean effect size - actual effect size. Pearson's correlations were performed to evaluate the correlation between effect size variation and sample sizes. For assessing outliers in all job-interview topic studies, the 95% CI of mean effect size was calculated by the following formula: 95% CI = *M* ± *t*_(a/2, n-1)_* *SD*, and where *M* = the mean of effect size, *SD* = the standard deviation of effect size.

Overall study heterogeneity was calculated to determine the model of meta-analysis. If the heterogeneity was significant (*I*^*2*^ > 50%, *p* < .050), the random-effects model was used; otherwise, a fixed-effects model was employed. Begg's test [46] and Egger's test [47] were conducted for detecting publication bias. Trim and Fill methods [48] were applied to estimate the missing studies contributing to publication bias. Sensitivity analysis was performed to evaluate the stability of included studies by excluding studies one by one.

The standardized mean difference (SMD) (i.e., Cohen's *d*) of salivary cortisol level between peak and baseline was reported as the effect size in each study. The following formula was used: $SMD=\frac{M_{peak}-M_{baseline}}{\sqrt{\frac{S{D^{2}}_{peak}\;+\;S{D^{2}}_{baseline}}{2}}}$, and where *M*_*peak*_ = the mean of peak salivary cortisol, *M*_*baseline*_ = the mean of baseline salivary cortisol, *SD*_*peak*_ = the standard deviation of peak salivary cortisol, *SD*_*baseline*_ = the standard deviation of baseline salivary cortisol.

All statistical analyses were completed in R 4.0.2 with the *meta* package. The significance threshold was set at *p* < .050 when only one test was performed and was adjusted by Bonferroni correction in the multivariate meta-regression analysis (*p* < .0125).

## 3 Results

### 3.1 Study characteristics

A total of 65 articles (76 sub-studies) met inclusion criteria, with 5171 participants (2040 females (39.45%) and 3131 males (60.55%)). Participants’ mean age was 23.88, ranging from 18.44 to 48.07. Among the 76 sub-studies, 28 had only female participants, and 48 had only male participants. Seventy-one studies used job interview as the speech topic and 5 used other topics. The sample sizes of studies ranged from 7 to 463.

The average effect size of salivary cortisol responses in all included studies as measured by SMD was 0.93, 95% CI = 0.82 to 1.04, and the effect size was significantly different from zero (*p* < .001), indicating that TSST could effectively induce large salivary cortisol responses. Effect size and primarily coded variables for each study are provided in Appendix C**. Study characteristics**.

### 3.2 Publication bias and sensitivity analysis

The heterogeneity of 76 included sub-studies was significant (*Q* = 469.55, *df* = 75, *p* < .001; *I*^*2*^ = 84.13%, *p* < .001). Thus, the meta-analysis was performed with a random-effects model. Egger's Test (*t* = 4.65, *df* = 74, *p* < .001) detected potential publication bias, while Begg's Test (*z* = 1.02, *p* = .307) did not. The Trim-and-fill method using symmetry assumptions and iterative approaches estimated that this meta-analysis had 32 missing studies (Appendix D**. Funnel plot**). The adjusted SMD was 0.55, 95% CI = 0.41 to 0.70, *p* < .001 under random-effects model, which was not significantly different from the original SMD (0.93, 95% CI = 0.82 to 1.04, *p* < .001), suggesting that the included studies had no considerable publication bias.

Sensitivity analysis was conducted by omitting studies one by one to determine how each study impacts the overall effect size. After omitting studies, the remaining combined SMD did not differ from the original one, suggesting the overall effect size was relatively stable (Appendix E**. Sensitivity analysis**).

### 3.3 The effects of sample size in studies using job interview topic

**Fig. 2:**
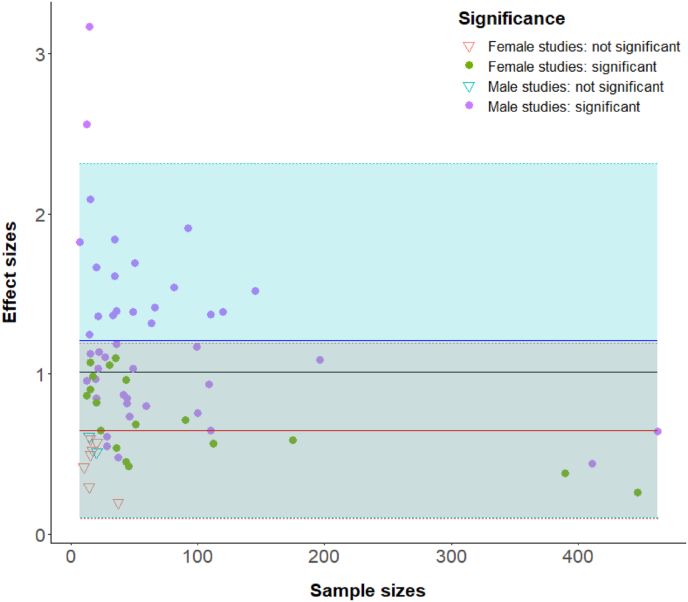
**The effect sizes reported by 71 sub-studies using job interview topic of salivary cortisol responses in TSST with various sample sizes.** The solid black line represents the mean effect size of all studies. The solid blue line represents the mean effect size of 46 male studies, and the blue fill represents 95%CI. The solid red line represents the mean effect size of 25 female studies, and the red fill represents 95%CI. Dash line for upper and lower boundaries of 95% CI. The significance indicates the differences of salivary cortisol levels between pre-task and post-task. (For interpretation of the references to colour in this figure legend, the reader is referred to the Web version of this article.)

We found that the small studies produced larger variations in the reported effect sizes than the large-sample studies(*r* = -0.24, *p* = .041), and the effect sizes can be frequently overestimated in these studies with a smaller sample (Fig. 2). Three studies (4.23%, 3/71) reported effect size as outliers in the job interview topic studies. They are all small sample studies. To make a conservative estimate of the minimum sample size needed for TSST study, we chose the lower confidence interval calculated by the meta-analysis as the threshold and used the strictest statistical parameters by G*Power 3.1. 40 sample is needed to reach statistical power as .99 with the two-tailed significance level of 0.01 and the effect size of 0.82. All nine job-interview topic studies with no significant findings of salivary cortisol concentration between pre-and post-tasks had fewer than 40 participants. Therefore, both the statistical power estimate and empirical data showed that TSST studies with about 40 samples should deliver sufficient statistical power to detect robust salivary cortisol responses.

### 3.4 The multivariate meta-regression model to predict salivary cortisol responses

We performed multivariate meta-regression to determine the impact of speech topics, sex, and the interaction between speech topics and sex on salivary cortisol response under the random-effects model. The regression analysis showed these variables could explain 42.68% of the variation salivary cortisol response and effectively account for the study heterogeneity (F(*df1* = 3, *df2* = 72) = 11.98, *p* < .001), see Table 1. Sex could significantly predict the salivary cortisol response (*p* < .00025). However, the effect of speech topics and the interaction between speech topics and sex were not significant after correcting for multiple testing in the regression model (all *p*-value > .0125).

**Table 1:** Contribution of the major variables to salivary cortisol response in TSST using multivariate meta-regression.

	Coefficient	*SE*	*z*	95% CI
**Speech topics**				
Job interview topic (reference group)				
Other speech topics	-0.56	0.31	-1.77	(-1.18, 0.07)
**Sex**				
Male (reference group)				
Female	-0.51	0.10	-5.13 ***	(-0.71, -0.31)
Speech topics * Sex				
Other speech topics: female	0.23	0.39	0.58	(-0.56, 1.01)
**Intercept**	1.13	0.06	19.23 ***	(1.01, 1.25)

*Notes:* The reference group was set for avoiding collinearity. The significance threshold was corrected by Bonferroni method. * indicates *p* < .0125, ** indicates *p* < .0025, *** indicates *p* < .00025.

### 3.5 The effects of sex on salivary cortisol responses

**Fig. 3:**
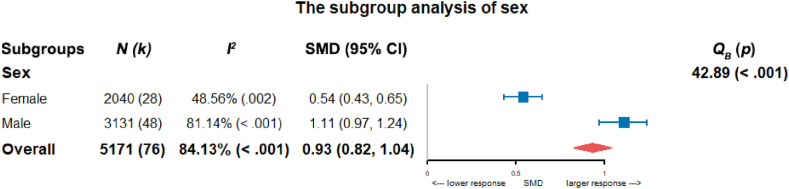
Forest plot of salivary cortisol response comparing sex groups in the meta-analysis with the random-effects model. *N* represents the sample sizes, and *k* represents the number of sub-studies. *I*^*2*^ represents the study heterogeneity. SMD represents the standardized mean difference, i.e., Cohen's *d*. *Q*_*B*_ represents inter-subgroup differences. Horizontal error bars represent 95% CI for effect sizes.

The above regression model showed that sex could significantly predict the salivary cortisol response, so we performed subgroup analysis of sex to identify the inter-group difference. The salivary cortisol responses in males (SMD = 1.11, 95% CI = 0.97 to 1.24) were higher than in females (SMD = 0.54, 95% CI = 0.43 to 0.65). Sex difference in salivary cortisol responses were significant (*Q*_*B*_ = 42.89, *df* = 1, *p* < .001), indicating that sex had a robust contribution to salivary cortisol response in TSST studies (Fig. 3).

We further evaluated the sex effects on salivary cortisol concentration at baseline and peak, respectively, to explore the source of sex differences on salivary cortisol responses. Significant sex differences in salivary cortisol concentrations were found at baseline (males: mean *M* = 8.61 nom/l, 95% CI = 7.72 to 9.51; females: *M* = 7.08 nom/l, 95% CI = 5.81 to 8.36; *t* = -2.03, *df* = 74, *p* = .046). Moreover, males had higher peak salivary cortisol concentration (*M* = 17.15 nom/l, 95% CI = 15.52 to 18.79) than females (*M* = 10.87 nom/l, 95% CI = 9.04 to 12.70) after completing the TSST (*t* = -4.96, *df* = 74, *p* < .001).

## 4 Discussion

The average effect size in 76 TSST sub-studies in this meta-analysis was 0.93, confirming that TSST could effectively induce salivary cortisol responses [11,17]. We quantitatively assessed the effects of sample size, speech topic, sex, and sex-speech topic interaction in this meta-analysis.

We concluded that 40 is the minimum number of samples to use in the TSST to study salivary cortisol responses. To our knowledge, this was the first evaluation of the effect of sample size on TSST results. Small studies frequently overestimated the effect sizes in publications, which is the well-known “winner's curse” phenomenon. The studies with more than 40 samples have sufficient statistical power to detect robust stress responses reporting significant salivary cortisol responses.

The effects of speech topics on salivary cortisol responses were evaluated in this meta-analysis with a large collection of TSST. We organized the studies into those with job interview as the speech topic and those with other speech topics to determine if there is a significant difference between job interview and modified speech topics. The salivary cortisol response of modified speech topics was not significantly different from that of the job interview topic. It should be noted that all studies using other topics had fewer than 40 samples, except for the study of Keenan et al. [32], and consequently are underpowered for evaluating their effects by each specific design individually. However, by combining experiments that used topics other than job interview, we were able to achieve a well-powered meta-analysis. The effect size of studies using the other topics was not significantly different from studies using job interview. Nonetheless, the effect variation (i.e., heterogeneity) of the other topics was small (Appendix F**. Forest plot**). The homogeneously smaller effect size from the other topics compared to job interview suggested that other topics would be unlikely to produce stronger effects than job interview. Therefore, we concluded that there is no significant difference of cortisol response in TSST studies using job interview and using other speech topics.

We speculated why the job interview and other speech topics had similar salivary cortisol responses. Mason [49] indicated that personal involvement was relevant to stress responses. When public speaking on a superficial, not self-relevant topic such as describing a holiday trip, a novel, or a movie, the degree of ego-involvement is low [50]. The participant-centered topics like job interview and accusation defense involve higher levels of ego-involvement and may induce stronger stress responses than the topics that focus on reading and summarizing a specific article [51]. All the topics in this study (i.e., job interview, accusation defense, self-introduction, and unfair treatment) are participant-centered, ego-involved. The speech topics tested did not cause different salivary cortisol responses. Their effect difference, if any, is too small to be detected in several hundreds of subjects. Since there is not currently a quantitative measure of ego-involvement, we cannot directly evaluate whether ego-involvement is similar among these topics.

We confirmed the sex differences of salivary cortisol responses in this meta-analysis. Males had stronger salivary cortisol responses than females in TSST studies, suggesting males and females react differently to stress. The possible explanation is that the HPA axis responses are prone to be higher in males than in females who are at the ages between puberty and menopause [37]. Moreover, the sex differences of salivary cortisol responses may result from different coping styles for distressing situations between males and females [38]. Males are prone to be stressed for situations involving intellectual inferiority and performance failures (e.g., public speaking and mental arithmetic), whereas females are particularly vulnerable when facing inconsistent commitments [52,53]. However, the interaction between sex and speech topics may be too small to be detected in this study.

We further found that sex differences occurred both at baseline before TSST and peak after exposure to TSST. It was reported that sex had significant effects on peak cortisol and continued through to recovery, but not on baseline cortisol, which is slightly different from our results [14]. Sex hormones can regulate cortisol levels, which could be an explanation of why the sex difference was significant [2,54]. Females in the follicular phase had lower cortisol levels than males, while females in the luteal phase showed similar cortisol levels to males [55,56]. Since most of the studies did not collect hormone information on participants, we could not evaluate the effects of sex hormones in this study. It is speculated that the females' salivary cortisol response will be more variable than males, and participants’ sex hormone status should be reported in future studies [2,10,37].

This meta-analysis should be viewed with limitations. Residual heterogeneity (i.e., intercept) had a significant contribution in the multivariate regression model, and the heterogeneity within subgroups was relatively high. This indicated that we missed other contributing variables on salivary cortisol responses in our data. In our meta-analysis with a large number of healthy adults, participants' age may be one of the contributors to cortisol responses. Previous studies suggested age effects on cortisol response, but there are conflicting findings in the literature [35,57,58]. Moreover, a significant interaction between age and sex was reported by Seeman et al. [59]. Participants’ age distribution of included studies is concentrated (mostly 20–30 years old), so we could not analyze the effects of age. More systematic assessments will be needed to understand other factors influencing the salivary cortisol response to stress. Another limitation is the sample distribution of speech topics. There is not enough power to evaluate the effects of each specific topic differed from job interview individually. Also, the speculation of ego-involvement levels of speech topics should be viewed cautiously.

## 5 Conclusions

The current meta-analysis confirms that the TSST effectively induces salivary cortisol responses. More importantly, we assessed the influence of several important factors on the outcomes of TSST as measured by salivary cortisol. The major findings include:

1. Forty samples is the minimum for detecting robust salivary cortisol responses.
2. The speech topics tested did not significantly alter salivary cortisol responses.
3. Sex differences in salivary cortisol reactivity were confirmed. Males had higher salivary cortisol levels than females in the TSST at both baseline and peak.

## Funding

The project is funded by Shaanxi Normal University internal fund. There is no grant from funding agencies in the public, commercial, or not-for-profit sectors.

## Declaration of competing interest

None.
