# Supplementary material for: A meta-analysis of salivary cortisol responses in the Trier Social Stress Test to evaluate the effects of speech topics, sex, and sample size
Source: Compr Psychoneuroendocrinol. 2022 Feb 10;10:100125. doi: 10.1016/j.cpnec.2022.100125 (PMC9216334; doi:10.1016/j.cpnec.2022.100125)
Supplement: Multimedia component 1 [file mmc1.docx]

# Supplementary data

# Appendix A. Search strategies

**Table A.1**

The executed search strategies in each database.

| **Database** | **Results returned** | **MeSH Terms and Entry Terms** | **Filters** |
| --- | --- | --- | --- |
| **PubMed** | 512 items | “Trier Social Stress Test”  “Hydrocortisone”  “Cortisol”  “Hydrocortisone, (9 beta,10 alpha,11 alpha)-Isomer”  “Hydrocortisone, (11 alpha)-Isomer”  “11-Epicortisol”  “11 Epicortisol”  “Epicortisol”  “Cortifair”  “Cortril” | “Journal Article”  “Abstract”  “From 1993/01/01 to 2020/12/31”  “Humans”  “Chinese or English”  “Adult: 19-44 years” |
| **PsycNET** | 828 items |  | “Journal Article”  “Empirical Study”  “Peer-Reviewed Journals”  “From 1993 to 2020”  “Human”  “Adulthood: 18 years & older” |
| **Web of Science** | 974 items |  | “Article”  “Chinese or English” |
| **CNKI** | 178 items | “特里尔社会应激测验”  “皮质醇” | “Chinese or English”  “From 1993/01/01 to 2020/12/31” |

*Notes:* These searches were conducted on April 21, 2020. CNKI = China National Knowledge Infrastructure.

# Appendix B. Quality assessment

**Table B.1**

The problems and score for all articles in quality assessment.

| **Study** | **Problems** | **Score** |
| --- | --- | --- |
| Aleknaviciute et al. (2016) | / | 100.00% |
| Bagley et al. (2011) | Small sample size^7^ | 95.45% |
| Brody (2002) | Question or objective were reported vaguely/incompletely ^1^; Small sample size ^7^ | 90.91% |
| Cantave et al. (2019) | Inclusion/exclusion criteria were not completely described ^3^; Imprecise results ^7^ | 90.91% |
| Carpenter et al. (2011) | / | 100.00% |
| Chopra et al. (2009) | Modest sample size ^7^ | 95.45% |
| Christian et al. (2013) | / | 100.00% |
| Clow et al. (1997) | Question or objective were reported vaguely/incompletely ^1^; Selection methods were not completely described ^3^; Insufficient relevant baseline/demographic information clearly characterizing the participants ^4^; Small sample size ^7^ | 79.17% |
| de Jong et al. (2015) | Question or objective were reported vaguely/incompletely ^1^; Inclusion/exclusion criteria were not completely described ^3^; Sufficient relevant baseline/demographic information clearly characterizing the participants ^4^; Random allocation not mentioned ^5^; Small sample size ^7^; Confounding not considered ^10^ | 70.83% |
| de Timary et al. (2008) | Question or objective were reported vaguely/incompletely ^1^; Insufficient relevant baseline/demographic information clearly characterizing the participants ^4^; Small sample size ^7^; Confounding not considered ^10^ | 86.36% |
| DeRijk et al. (2006) | Question or objective were reported vaguely/incompletely ^1^; Selection methods were not completely described ^3^; Insufficient relevant baseline/demographic information clearly characterizing the participants ^4^ | 86.36% |
| Domes et al. (2004) | / | 100.00% |
| Edelstein et al. (2010) | Selection methods were not completely described ^3^; Insufficient relevant baseline/demographic information clearly characterizing the participants ^4^; Analytic methods are not reported ^8^ | 86.36% |
| Engert et al. (2011) | Confounding not considered ^10^ | 95.45% |
| Fischer et al. (2019) | Insufficient relevant baseline/demographic information clearly characterizing the participants ^4^; Conclusions were reported incompletely ^12^ | 91.67% |
| Gaab et al. (2005) | Question or objective were reported vaguely/incompletely ^1^; Conclusions were reported vaguely ^12^ | 90.91% |
| Gideon et al. (2019) | Question or objective were reported vaguely/incompletely ^1^ | 95.83% |
| Gröpel et al. (2018) | / | 100.00% |
| Guo et al. (2017) | Selection methods were not completely described ^3^ | 95.45% |
| Hidalgo et al. (2015) | Insufficient relevant baseline/demographic information clearly characterizing the participants ^4^ | 95.83% |
| Huang et al. (2015) | / | 100.00% |
| Jackowska et al. (2018) | Confounding not considered ^10^ | 95.45% |
| Keenan et al. (2021) | Question or objective were reported vaguely/incompletely ^1^; Selection methods were not completely described ^3^; Insufficient relevant baseline/demographic information clearly characterizing the participants ^4^; Analytic methods were not reported ^8^ | 81.82% |
| Kennedy et al. (2014) | / | 100.00% |
| Kirschbaum et al. (1995) | Question or objective were reported vaguely/incompletely ^1^; Small sample size ^7^; | 90.91% |
| Kudielka et al. (2000) | Question or objective were reported vaguely/incompletely ^1^; Small sample size ^7^; Confounding not considered ^10^ | 86.36% |
| Kumsta et al. (2013) | Selection methods were not completely described ^3^; Insufficient relevant baseline/demographic information clearly characterizing the participants ^4^ | 90.91% |
| Langer et al. (2019) | Question or objective were reported vaguely/incompletely ^1^; Insufficient relevant baseline/demographic information clearly characterizing the participants ^4^; Small sample size ^7^ | 87.50% |
| Luethi et al. (2009) | Selection methods were not completely described ^3^; Insufficient relevant baseline/demographic information clearly characterizing the participants ^4^; Small sample size ^7^ | 87.50% |
| Mahon et al. (2013) | Question or objective were reported vaguely/incompletely ^1^; Insufficient relevant baseline/demographic information clearly characterizing the participants ^4^ | 90.91% |
| Massar et al. (2017) | Selection methods were not completely described ^3^; Insufficient relevant baseline/demographic information clearly characterizing the participants ^4^ | 95.45% |
| Mikolajczak et al. (2008) | Insufficient relevant baseline/demographic information clearly characterizing the participants ^4^; Small sample size ^7^; Confounding not considered ^10^ | 86.36% |
| Monteleone et al. (2011) | Question or objective were reported vaguely/incompletely ^1^; Selection methods were not completely described ^3^; Small sample size ^7^ | 86.36% |
| Monteleone et al. (2012) | Question or objective were reported vaguely/incompletely ^1^; Selection methods were not completely described ^3^; Small sample size ^7^ | 86.36% |
| Monteleone et al. (2018) | Selection methods were not completely described ^3^; Small sample size ^7^ | 90.91% |
| Nater et al. (2010) | Small sample size ^7^ | 95.45% |
| O’Leary et al. (2007) | Analytic methods were not reported ^8^ | 95.45% |
| Petrowski and Conrad (2019) | Selection methods were not completely described ^3^; Small sample size ^7^ | 90.91% |
| Prall et al. (2017) | Question or objective were reported vaguely/incompletely ^1^; Small sample size ^7^ | 90.91% |
| Rimmele et al. (2007) | / | 100.00% |
| Rimmele et al. (2009) | Question or objective were reported vaguely/incompletely ^1^ | 95.45% |
| Schlotz et al. (2011) | Question or objective were reported vaguely/incompletely ^1^; Small and restricted sample ^7^ | 90.91% |
| Scholz et al. (2009) | Selection methods were not completely described ^3^; Insufficient relevant baseline/demographic information clearly characterizing the participants ^4^; Confounding not considered ^10^ | 87.50% |
| Sep et al. (2019) | Selection methods were not completely described ^3^ | 95.83% |
| Shalev et al. (2009) | Question or objective were reported vaguely/incompletely ^1^; Selection methods were not completely described ^3^; Conclusions were reported vaguely ^12^ | 86.36% |
| Shiban et al. (2016) | Insufficient relevant baseline/demographic information clearly characterizing the participants ^4^; Small sample size ^7^; Confounding not considered ^10^ | 87.50% |
| Shirotsuki et al. (2009) | Small sample size ^7^ | 95.45% |
| Shirotsuki et al. (2020) | Question or objective were reported vaguely/incompletely ^1^; Confounding not considered ^10^ | 90.91% |
| Stephens et al. (2016) | / | 100.00% |
| Strahler et al. (2015) | Small sample size ^7^; Confounding not considered ^10^ | 90.91% |
| Suárez-Hitz et al. (2012) | Question or objective were reported vaguely/incompletely ^1^; | 95.45% |
| Thoma et al. (2017) | / | 100.00% |
| Trautmann et al. (2018) | Question or objective were reported vaguely/incompletely ^1^; Selection methods were not completely described ^3^; Random allocation not mentioned ^5^ | 87.50% |
| van Ast et al. (2014) | Question or objective were reported vaguely/incompletely ^1^; Selection methods were not completely described ^3^; Insufficient relevant baseline/demographic information clearly characterizing the participants ^4^ | 87.50% |
| van Leeuwen et al. (2019) | Small sample size ^7^ | 95.83% |
| Villada et al. (2017) | Selection methods were not completely described ^3^; Small sample size ^7^; Confounding not considered ^10^ | 86.36% |
| Wieck et al. (2013) | Question or objective were reported vaguely/incompletely ^1^; Small sample size ^7^ | 90.91% |
| Wilson et al. (2015) | Inclusion/exclusion criteria were not completely described ^3^ | 95.45% |
| Wingenfeld et al. (2018) | Insufficient relevant baseline/demographic information clearly characterizing the participants ^4^; Small and restricted sample ^7^ | 91.67% |
| Wirtz et al. (2007) | Question or objective were reported vaguely/incompletely ^1^; Selection methods were not completely described ^3^ | 90.91% |
| Wolf (2012) | Random allocation not mentioned ^5^; Confounding not considered ^10^ | 91.67% |
| Wolf et al. (2015) | Selection methods were not completely described ^3^; Insufficient relevant baseline/demographic information clearly characterizing the participants ^4^; Confounding not considered ^10^ | 87.50% |
| Zandara et al. (2018) | / | 100.00% |
| Zhu et al. (2016) | Question or objective were reported vaguely/incompletely ^1^ | 95.45% |
| Zimmer et al. (2019) | / | 100.00% |

*Notes:* According to the Checklist for assessing the quality of quantitative studies of QualSyst, all 65 articles were assessed. ^1^ refers to “Question or objective sufficiently described?”. ^2^ refers to “Design evident and appropriate to answer study question?”. ^3^ refers to “Method of subject selection (and comparison group selection, if applicable) or source of information/input variables (e.g., for decision analysis) is described and appropriate.” ^4^ refers to “Subject (and comparison group, if applicable) characteristics or input variables/information (e.g., for decision analyses) sufficiently described?”. ^5^ refers to “If random allocation to treatment group was possible, is it described?”, if applicable. ^6^ refers to “Outcome and (if applicable) exposure measure(s) well defined and robust to measurement/misclassification bias? Means of assessment reported?”. ^7^ refers to “Sample size appropriate?” .^8^ refers to “Analysis described and appropriate?”. ^9^ refers to “Some estimate of variance (e.g., confidence intervals, standard errors) is reported for the main results/outcomes (i.e., those directly addressing the study question/objective upon which the conclusions are based)?”. ^10^ refers to “Controlled for confounding?”. ^11^ refers to “Results reported in sufficient detail?”. ^12^ refers to “Do the results support the conclusions?”. All criteria were scored depending on the degree of meet (“yes” = 2, “partial” = 1, “no” = 0). Special criteria were marked “n/a”, if not applicable. Quality score = total sum / total possible sum, and where total sum = (number of “yes” * 2) + (number of “partial” * 1), total possible sum = 28 - (number of “n/a” * 2).

# Appendix C. Study characteristics

**Table C.1**

Coded variables and effect sizes (SMD) for the studies included in the meta-analysis.

| **Study** | **Type of speech topic** | **Sex** | ***N*** | **Mean age (years)** | **SMD** | **mean salivary cortisol (SD)** | |
| --- | --- | --- | --- | --- | --- | --- | --- |
|  |  |  |  |  |  | **baseline** | **peak** |
| Aleknaviciute et al. (2016) | job interview | female | 35 | 28.60 | 1.10 | 9.06 (4.84) | 18.92 (11.73) |
| Bagley et al. (2011): sub-study 1 | defending against an accusation of shoplifting ^b^ | male | 22 | 32.40 | 0.35 | 13.23 (7.41) | 15.85 (7.41) |
| Bagley et al. (2011): sub-study 2 | defending against an accusation of shoplifting ^b^ | female | 14 | 31.80 | 0.41 | 8.56 (4.15) | 10.58 (5.65) |
| Brody (2002): sub-study 1 | job interview | male | 36 | 24.48 | 1.39 | 12.18 (7.03) | 26.79 (13.07) |
| Brody (2002): sub-study 2 | job interview | female | 43 | 24.78 | 0.96 | 11.18 (6.06) | 19.78 (11.09) |
| Cantave et al. (2019) | job interview | male | 100 | 24.10 | 0.76 | 4.14 (2.76) | 7.45 (5.52) |
| Carpenter et al. (2011) | job interview | female | 90 | 29.20 | 0.71 | 6.46 (5.12) | 11.84 (9.39) |
| Chopra et al. (2009): sub-study 1 | job interview | male | 14 | 39.40 | 1.25 | 10.97 (5.80) | 19.70 (8.02) |
| Chopra et al. (2009): sub-study 2 | job interview | female | 14 | 39.40 | 0.29 | 11.63 (8.60) | 14.47 (10.57) |
| Christian et al. (2013) | job interview | female | 37 | 24.03 | 0.20 | 5.52 (3.59) | 6.35 (4.69) |
| Clow et al. (1997) | job interview | male | 14 | 21.00 | 0.61 | 10.86 (9.35) | 18.48 (14.93) |
| de Jong et al. (2015): sub-study 1 | job interview | male | 15 | 24.00 | 2.09 | 3.09 (1.67) | 19.15 (10.73) |
| de Jong et al. (2015): sub-study 2 | job interview | female | 15 | 23.00 | 1.07 | 3.94 (1.67) | 9.41 (7.01) |
| de Timary et al. (2008) | job interview | male | 28 | 20.86 | 0.61 | 8.57 (4.55) | 12.00 (6.56) |
| DeRijk et al. (2006) | job interview | male | 110 | 18.70 | 0.65 | 13.02 (10.50) | 20.84 (13.46) |
| Domes et al. (2004) | job interview | male | 20 | 27.80 | 0.85 | 10.15 (5.72) | 16.84 (9.57) |
| Edelstein et al. (2010): sub-study 1 | introducing one’s self to new classmates ^b^ | male | 23 | 20.57 | 0.80 | 5.19 (5.43) | 11.16 (9.08) |
| Edelstein et al. (2010): sub-study 2 | introducing one’s self to new classmates ^b^ | female | 25 | 20.57 | 0.44 | 3.20 (2.72) | 4.95 (4.86) |
| Engert et al. (2011) | job interview | male | 49 | 22.41 | 1.03 | 3.48 (2.10) | 7.70 (5.39) |
| Fischer et al. (2019) | job interview | male | 63 | 24.00 | 1.32 | 11.23 (5.89) | 24.30 (12.73) |
| Gaab et al. (2005) | job interview | male | 81 | 24.60 | 1.54 | 13.18 (7.92) | 33.58 (17.01) |
| Gideon et al. (2019) | job interview | male | 34 | 23.24 | 1.84 | 5.72 (3.36) | 17.88 (8.71) |
| Gröpel et al. (2018) | job interview | male | 33 | 23.80 | 1.37 | 7.46 (3.49) | 16.67 (8.86) |
| Guo et al. (2017): sub-study 1 | job interview | male | 463 | 19.20 | 0.64 | 4.78 (2.92) | 7.36 (4.86) |
| Guo et al. (2017): sub-study 2 | job interview | female | 447 | 19.20 | 0.26 | 4.55 (4.10) | 5.72 (4.86) |
| Hidalgo et al. (2015) | job interview | male | 14 | 22.50 | 3.17 | 6.21 (2.96) | 20.42 (5.61) |
| Huang et al. (2015) | job interview | female | 36 | 22.03 | 0.54 | 13.85 (5.49) | 18.50 (10.96) |
| Jackowska et al. (2018) | job interview | male | 120 | 46.03 | 1.39 | 9.83 (6.93) | 23.63 (12.22) |
| Keenan et al. (2021) | imagining experiencing unfair treatment due to personal attributes ^b^ | female | 274 | 24.20 | 0.15 | 3.45 (2.43) | 3.87 (3.09) |
| Kennedy et al. (2014) | job interview | female | 15 | 23.30 | 0.60 | 2.37 (2.41) | 4.85 (5.37) |
| Kirschbaum et al. (1995) | job interview | female | 12 | 23.15 | 0.87 | 7.40 (5.54) | 11.30 (3.12) |
| Kudielka et al. (2000) | job interview | male | 12 | 25.60 | 0.96 | 7.78 (3.57) | 14.78 (9.70) |
| Kumsta et al. (2013) | job interview | male | 196 | 23.70 | 1.09 | 9.35 (6.58) | 18.17 (9.40) |
| Langer et al. (2019) | job interview | male | 36 | 24.38 | 1.19 | 9.90 (7.16) | 20.97 (11.09) |
| Luethi et al. (2009) | job interview | male | 19 | 23.40 | 0.97 | 10.00 (3.90) | 15.60 (7.2) |
| Mahon et al. (2013) | job interview | female | 175 | 23.00 | 0.59 | 8.00 (4.14) | 11.86 (8.28) |
| Massar et al. (2017) | job interview | male | 59 | 22.83 | 0.80 | 6.04 (3.30) | 9.04 (4.15) |
| Mikolajczak et al. (2008) | job interview | male | 28 | 20.86 | 0.55 | 8.49 (5.50) | 11.90 (6.79) |
| Monteleone et al. (2011) | job interview | female | 15 | 23.60 | 0.50 | 7.14 (8.29) | 10.93 (6.93) |
| Monteleone et al. (2012) | job interview | female | 10 | 24.10 | 0.42 | 7.82 (6.99) | 11.76 (11.23) |
| Monteleone et al. (2018) | job interview | female | 17 | 26.00 | 0.99 | 4.79 (2.19) | 8.33 (4.58) |
| Nater et al. (2010) | job interview | female | 17 | 27.20 | 0.52 | 11.91 (12.74) | 18.40 (12.12) |
| O’Leary et al. (2007): sub-study 1 | job interview | male | 41 | 18.51 | 0.87 | 8.29 (4.46) | 14.58 (9.19) |
| O’Leary et al. (2007): sub-study 2 | job interview | female | 43 | 18.44 | 0.45 | 6.49 (2.53) | 8.16 (4.61) |
| Petrowski and Conrad (2019) | job interview | male | 7 | 31.13 | 1.82 | 4.60 (1.69) | 17.35 (9.75) |
| Prall et al. (2017) | job interview | male | 27 | 21.60 | 1.10 | 4.69 (2.21) | 9.93 (6.35) |
| Rimmele et al. (2007) | job interview | male | 44 | 21.67 | 0.82 | 12.62 (6.60) | 21.93 (14.69) |
| Rimmele et al. (2009) | job interview | male | 92 | 24.39 | 1.91 | 8.65 (4.32) | 22.22 (9.08) |
| Schlotz et al. (2011) | job interview | male | 66 | 24.80 | 1.41 | 13.90 (6.09) | 27.60 (12.27) |
| Scholz et al. (2009) | job interview | male | 21 | 25.73 | 1.03 | 7.62 (5.20) | 15.10 (8.83) |
| Sep et al. (2019) | job interview | male | 34 | 24.01 | 1.61 | 10.37 (5.32) | 18.76 (5.08) |
| Shalev et al. (2009): sub-study 1 | job interview | male | 46 | 25.20 | 0.73 | 8.18 (5.83) | 12.36 (5.56) |
| Shalev et al. (2009): sub-study 2 | job interview | female | 51 | 23.51 | 0.69 | 6.46 (1.79) | 7.79 (2.07) |
| Shiban et al. (2016) | job interview | male | 15 | 23.76 | 1.12 | 5.48 (4.65) | 11.84 (6.51) |
| Shirotsuki et al. (2009) | job interview | male | 22 | 21.62 | 1.14 | 5.94 (3.48) | 11.35 (5.76) |
| Shirotsuki et al. (2020) | job interview | male | 44 | 21.52 | 0.85 | 8.13 (4.33) | 15.43 (11.38) |
| Stephens et al. (2016): sub-study 1 | job interview | male | 99 | 23.60 | 1.17 | 5.24 (2.75) | 12.42 (8.24) |
| Stephens et al. (2016): sub-study 2 | job interview | female | 112 | 23.60 | 0.57 | 5.52 (5.84) | 8.83 (5.84) |
| Strahler et al. (2015) | job interview | male | 20 | 28.25 | 0.51 | 11.53 (9.35) | 16.13 (8.54) |
| Suárez-Hitz et al. (2012) | job interview | female | 20 | 29.00 | 0.82 | 5.34 (2.91) | 10.23 (7.92) |
| Thoma et al. (2017) | job interview | male | 110 | 46.00 | 1.37 | 7.36 (4.61) | 19.28 (11.37) |
| Trautmann et al. (2018) | job interview | male | 145 | 28.60 | 1.52 | 10.01 (5.10) | 24.40 (12.40) |
| van Ast et al. (2014) | job interview | female | 20 | 22.00 | 0.57 | 10.95 (4.83) | 15.02 (8.77) |
| van Leeuwen et al. (2019) | job interview | male | 20 | 39.00 | 1.66 | 7.33 (3.09) | 14.04 (4.79) |
| Villada et al. (2017) | job interview | female | 30 | 18.73 | 1.06 | 4.48 (3.48) | 12.19 (9.71) |
| Wieck et al. (2013) | job interview | female | 15 | 48.07 | 0.90 | 5.20 (2.36) | 10.20 (7.47) |
| Wilson et al. (2015) | job interview | male | 109 | 22.42 | 0.94 | 5.38 (2.90) | 10.70 (7.48) |
| Wingenfeld et al. (2018) | job interview | female | 23 | 30.20 | 0.65 | 1.23 (1.06) | 2.10 (1.58) |
| Wirtz et al. (2007) | job interview | male | 50 | 42.50 | 1.69 | 7.42 (4.02) | 21.31 (10.89) |
| Wolf (2012) | job interview | male | 12 | 24.00 | 2.56 | 10.64 (3.05) | 24.23 (6.86) |
| Wolf et al. (2015) | job interview | male | 49 | 24.40 | 1.39 | 9.98 (5.53) | 20.90 (9.66) |
| Zandara et al. (2018): sub-study 1 | job interview | male | 37 | 25.81 | 0.48 | 13.87 (4.68) | 16.74 (7.12) |
| Zandara et al. (2018): sub-study 2 | job interview | female | 45 | 24.31 | 0.42 | 9.07 (4.83) | 11.62 (7.04) |
| Zhu et al. (2016): sub-study 1 | job interview | male | 411 | 20.00 | 0.44 | 15.40 (10.30) | 20.20 (11.40) |
| Zhu et al. (2016): sub-study 2 | job interview | female | 390 | 20.10 | 0.38 | 12.80 (9.30) | 16.50 (10.20) |
| Zimmer et al. (2019) | job interview | male | 21 | 26.05 | 1.36 | 5.87 (4.03) | 14.29 (7.75) |

*Notes:* In the same articles, sub-study 1 provided males’ data, and sub-study 2 females’ data. In the column of type of speech topics, a refers other speech topics differed from job interview topic. *N* represents sample sizes. SMD represents the standardized mean difference, i.e., Cohen’s *d*.

# Appendix D. Funnel plot


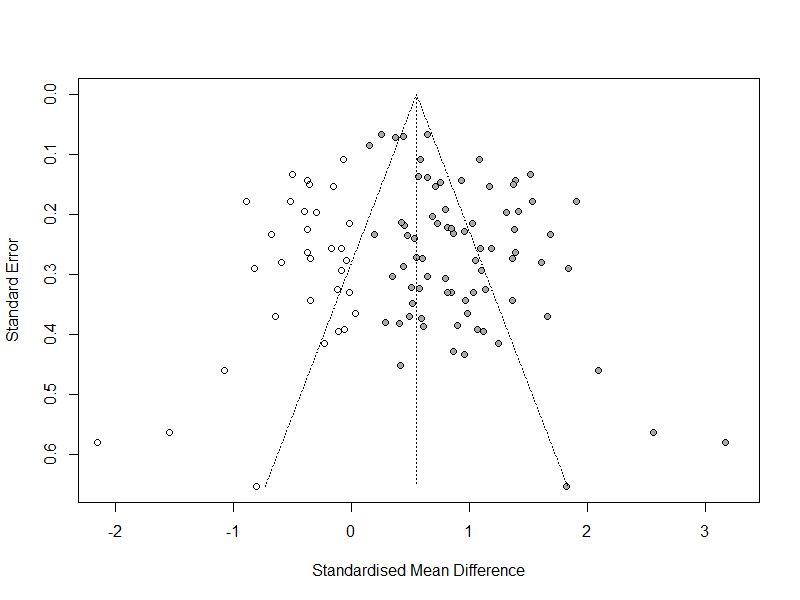


**Fig. D.1.** Funnel plot. Solid circles represent original studies, and open circles represent imputed missing studies using Trim and Fill methods.

# Appendix E. Sensitivity analysis


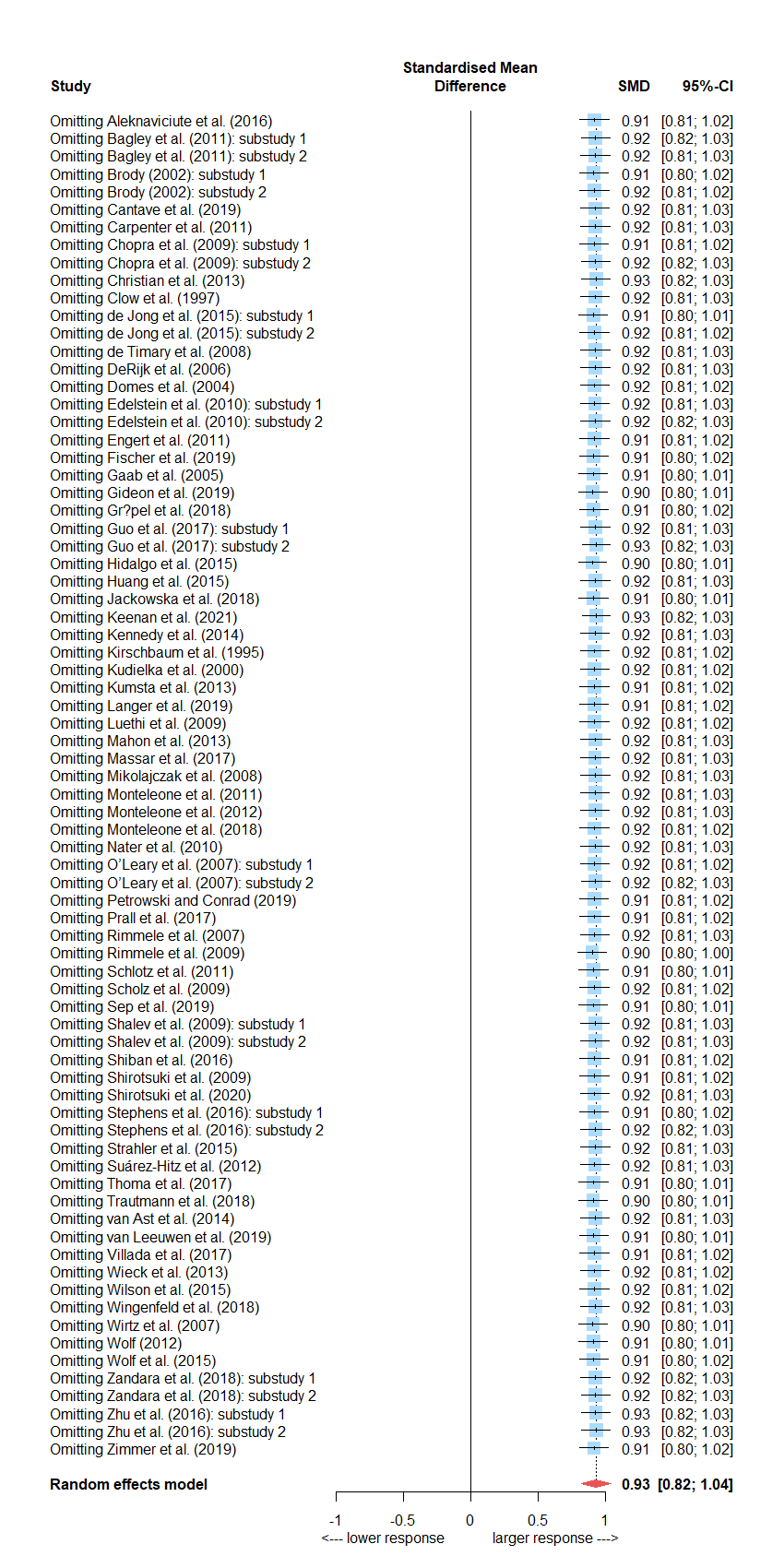


**Fig. E.1.** Sensitivity analysis by omitting study one by one under the random-effects model. Sub-study 1 or 2 refers to the males’ or females’ data from one article.

# Appendix F. Forest plot


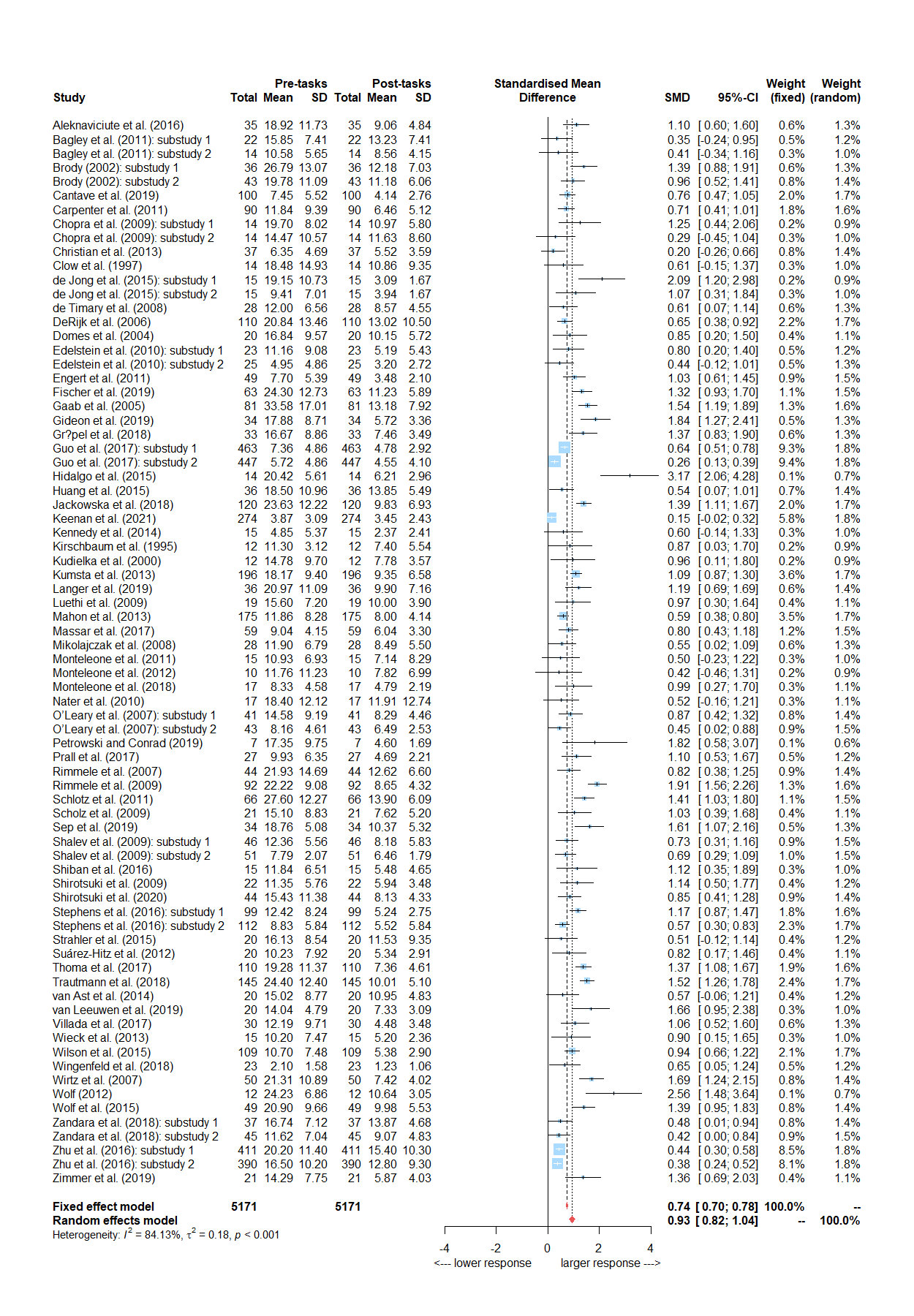


**Fig. F.1.** Forest plot of salivary cortisol response comparing studies using job interview and studies using other speech topics in the meta-analysis with the random-effects model. *I^2^* represents the study heterogeneity. SMD represents the standardized mean difference, i.e., Cohen’s *d*.

# References (articles included in the meta analysis)

Aleknaviciute, J., Tulen, J. H., Kamperman, A. M., de Rijke, Y. B., Kooiman, C. G., Kushner, S. A., 2016. Borderline and cluster C personality disorders manifest distinct physiological responses to psychosocial stress. Psychoneuroendocrinology. 72. 131–138. https://doi.org/10.1016/j.psyneuen.2016.06.010.

Bagley, S. L., Weaver, T. L., Buchanan, T. W., 2011. Sex differences in physiological and affective responses to stress in remitted depression. Physiology & Behavior. 104(2). 180–186. https://doi.org/10.1016/j.physbeh.2011.03.004.

Brody, S., 2002. Age at first intercourse is inversely related to female cortisol stress reactivity. Psychoneuroendocrinology. 27(8). 933–943. https://doi.org/10.1016/S0306-4530(02)00007-0.

Cantave, C. Y., Langevin, S., Marin, M., Brendgen, M., Lupien, S., Ouellet-Morin, I., 2019. Impact of maltreatment on depressive symptoms in young male adults: The mediating and moderating role of cortisol stress response and coping strategies. Psychoneuroendocrinology. 103. 41–48. https://doi.org/10.1016/j.psyneuen.2018.12.235.

Carpenter, L. L., Shattuck, T. T., Tyrka, A. R., Geracioti, T. D., Price, L. H., 2011. Effect of childhood physical abuse on cortisol stress response. Psychopharmacology. 214(1). 367–375. https://doi.org/10.1007/s00213-010-2007-4.

Chopra, K. K., Ravindran, A., Kennedy, S. H., Mackenzie, B., Matthews, S., Anisman, H., Bagby, R. M., Farvolden, P., Levitan, R. D., 2009. Sex differences in hormonal responses to a social stressor in chronic major depression. Psychoneuroendocrinology. 34(8). 1235–1241. https://doi.org/10.1016/j.psyneuen.2009.03.014.

Christian, L. M., Glaser, R., Porter, K., Iams, J. D., 2013. Stress-Induced Inflammatory Responses in Women: Effects of Race and Pregnancy. Psychosomatic Medicine. 75(7). 658–669. https://doi.org/10.1097/PSY.0b013e31829bbc89.

Clow, A., Patel, S., Najafi, M., Evans, P. D., Hucklebridge, F., 1997. The cortisol response to psychological challenge is preceded by a transient rise in endogenous inhibitor of monoamine oxidase. Life Sciences. 61(5). 567–575. https://doi.org/0.1016/S0024-3205(97)00416-5.

de Jong, T. R., Menon, R., Bludau, A., Grund, T., Biermeier, V., Klampfl, S. M., Jurek, B., Bosch, O. J., Hellhammer, J., Neumann, I. D., 2015. Salivary oxytocin concentrations in response to running, sexual self-stimulation, breastfeeding and the TSST: The Regensburg Oxytocin Challenge (ROC) study. Psychoneuroendocrinology. 62. 381–388. https://doi.org/10.1016/j.psyneuen.2015.08.027.

de Timary, P., Roy, E., Luminet, O., Fillée, C., Mikolajczak, M., 2008. Relationship between alexithymia, alexithymia factors and salivary cortisol in men exposed to a social stress test. Psychoneuroendocrinology. 33(8). 1160–1164. https://doi.org/10.1016/j.psyneuen.2008.06.005.

DeRijk, R. H., Wüst, S., Meijer, O. C., Zennaro, M. C., Federenko, I. S., Hellhammer, D. H., Giacchetti, G., Vreugdenhil, E., Zitman, F. G., de Kloet, E. R., 2006. A common polymorphism in the mineralocorticoid receptor modulates stress responsiveness. The Journal of Clinical Endocrinology & Metabolism. 91(12). 5083–5089. https://doi.org/10.1210/jc.2006-0915.

Domes, G., Heinrichs, M., Rimmele, U., Reichwald, U., Hautzinger, M., 2004. Acute stress impairs recognition for positive words--association with stress-induced cortisol secretion. Stress. 7(3). 173–181. https://doi.org/10.1080/10253890412331273213.

Edelstein, R. S., Yim, I. S., Quas, J. A., 2010. Narcissism predicts heightened cortisol reactivity to a psychosocial stressor in men. Journal of Research in Personality. 44(5). 565–572. https://doi.org/10.1016/j.jrp.2010.06.008.

Engert, V., Vogel, S., Efanov, S. I., Duchesne, A., Corbo, V., Ali, N., Pruessner, J. C., 2011. Investigation into the cross-correlation of salivary cortisol and alpha-amylase responses to psychological stress. Psychoneuroendocrinology. 36(9). 1294–1302. https://doi.org/10.1016/j.psyneuen.2011.02.018.

Fischer, S., Spoerri, C. M., Gmuer, A., Wingeier, M., Nater, U. M., Gaab, J., Ehlert, U., Ditzen, B., 2019. Psychobiological impact of speaking a second language in healthy young men. Stress. 1–5. https://doi.org/10.1080/10253890.2019.1575805.

Gaab, J., Rohleder, N., Nater, U. M., Ehlert, U., 2005. Psychological determinants of the cortisol stress response: the role of anticipatory cognitive appraisal. Psychoneuroendocrinology. 30(6). 599–610. https://doi.org/10.1016/j.psyneuen.2005.02.001.

Gideon, A., Sauter, C., Fieres, J., Berger, T., Renner, B., Wirtz, P. H., 2019. Kinetics and Interrelations of the Renin Aldosterone Response to Acute Psychosocial Stress: A Neglected Stress System. The Journal of Clinical Endocrinology & Metabolism. 105(3). e762–e773. https://doi.org/10.1210/clinem/dgz190.

Gröpel, P., Urner, M., Pruessner, J. C., Quirin, M., 2018. Endurance- and Resistance-Trained Men Exhibit Lower Cardiovascular Responses to Psychosocial Stress Than Untrained Men. Frontiers in Psychology. 9. 852. https://doi.org/10.3389/fpsyg.2018.00852.

Guo, J., Mrug, S., Knight, D. C., 2017. Emotion socialization as a predictor of physiological and psychological responses to stress. Physiology & Behavior. 175. 119–129. https://doi.org/10.1016/j.physbeh.2017.03.046.

Hidalgo, V., Pulopulos, M. M., Puig-Perez, S., Espin, L., Gomez-Amor, J., Salvador, A., 2015. Acute stress affects free recall and recognition of pictures differently depending on age and sex. Behavioural Brain Research. 292. 393–402. https://doi.org/10.1016/j.bbr.2015.07.011.

Huang, Y., Zhou, R., Wu, M., Wang, Q., Zhao, Y., 2015. Premenstrual syndrome is associated with blunted cortisol reactivity to the TSST. Stress. 18(2). 160–168. https://doi.org/10.3109/10253890.2014.999234.

Jackowska, M., Fuchs, R., Klaperski, S., 2018. The association of sleep disturbances with endocrine and perceived stress reactivity measures in male employees. British Journal of Psychology. 109(1). 137–155. https://doi.org/10.1111/bjop.12250.

Keenan, K., Berona, J., Hipwell, A. E., Stepp, S. D., Romito, M. T., 2021. Validity of the Trier Social Stress Test in studying discrimination stress. Stress. 24(1). 113–119. https://doi.org/10.1080/10253890.2020.1741545.

Kennedy, P. J., Cryan, J. F., Quigley, E. M. M., Dinan, T. G., Clarke, G., 2014. A sustained hypothalamic-pituitary-adrenal axis response to acute psychosocial stress in irritable bowel syndrome. Psychological Medicine. 44(14). 3123–3134. https://doi.org/10.1017/S003329171400052X.

Kirschbaum, C., Pirke, K. M., Hellhammer, D. H., 1995. Preliminary evidence for reduced cortisol responsivity to psychological stress in women using oral contraceptive medication. Psychoneuroendocrinology. 20(5). 509–514. https://doi.org/10.1016/0306-4530(94)00078-O.

Kudielka, B. M., Schmidt-Reinwald, A. K., Hellhammer, D. H., Schürmeyer, T., Kirschbaum, C., 2000. Psychosocial Stress and HPA Functioning: No Evidence for a Reduced Resilience in Healthy Elderly Men. Stress. 3(3). 229–240. https://doi.org/10.3109/10253890009001127.

Kumsta, R., Chen, F. S., Pape, H. C., Heinrichs, M., 2013. Neuropeptide S receptor gene is associated with cortisol responses to social stress in humans. Biological Psychology. 93(2). 304–307. https://doi.org/10.1016/j.biopsycho.2013.02.018.

Langer, K., Moser, D., Otto, T., Wolf, O. T., Kumsta, R., 2019. Cortisol modulates the engagement of multiple memory systems: Exploration of a common NR3C2 polymorphism. Psychoneuroendocrinology. 107. 133–140. https://doi.org/10.1016/j.psyneuen.2019.05.006.

Luethi, M., Meier, B., Sandi, C., 2009. Stress Effects on Working Memory, Explicit Memory, and Implicit Memory for Neutral and Emotional Stimuli in Healthy Men. Frontiers in Behavioral Neuroscience. 2. 5. https://doi.org/10.3389/neuro.08.005.2008.

Mahon, P. B., Zandi, P. P., Potash, J. B., Nestadt, G., Wand, G. S., 2013. Genetic association of FKBP5 and CRHR1 with cortisol response to acute psychosocial stress in healthy adults. Psychopharmacology. 227(2). 231–241. https://doi.org/10.1007/s00213-012-2956-x.

Massar, S., Liu, J., Muhammad, N. B., Chee, M., 2017. Poor habitual sleep efficiency is associated with increased cardiovascular and cortisol stress reactivity in men. Psychoneuroendocrinology. 81. 151–156. https://doi.org/10.1016/j.psyneuen.2017.04.013.

Mikolajczak, M., Roy, E., Luminet, O., De Timary, P., 2008. Resilience and hypothalamic-pituitary-adrenal axis reactivity under acute stress in young men. Stress. 11(6). 477–482. https://doi.org/10.1080/10253890701850262.

Monteleone, A. M., Patriciello, G., Ruzzi, V., Cimino, M., Del Giorno, C., SteardoJr, L., Monteleoneb, P., Maj, M., 2018. Deranged emotional and cortisol responses to a psychosocial stressor in anorexia nervosa women with childhood trauma exposure: Evidence for a "maltreated ecophenotype"? Journal of Psychiatric Research. 104. 39–45. https://doi.org/10.1016/j.jpsychires.2018.06.013.

Monteleone, P., Scognamiglio, P., Canestrelli, B., Serino, I., Monteleone, A. M., Maj, M., 2011. Asymmetry of salivary cortisol and α-amylase responses to psychosocial stress in anorexia nervosa but not in bulimia nervosa. Psychological Medicine. 41(9). 1963–1969. https://doi.org/10.1017/S0033291711000092.

Monteleone, P., Tortorella, A., Scognamiglio, P., Serino, I., Monteleone, A. M., Maj, M., 2012. The Acute Salivary Ghrelin Response to a Psychosocial Stress Is Enhanced in Symptomatic Patients with Bulimia Nervosa: A Pilot Study. Neuropsychobiology. 66(4). 230–236. https://doi.org/10.1159/000341877.

Nater, U. M., Bohus, M., Abbruzzese, E., Ditzen, B., G Aab , J., Kleindienst, N., Ebner-Priemer, U., Mauchnik, J., Ehlert, U., 2010. Increased psychological and attenuated cortisol and alpha-amylase responses to acute psychosocial stress in female patients with borderline personality disorder. Psychoneuroendocrinology. 35(10). 1565–1572. https://doi.org/10.1016/j.psyneuen.2010.06.002.

O’Leary, M. M., Loney, B. R., Eckel, L. A., 2007. Gender differences in the association between psychopathic personality traits and cortisol response to induced stress. Psychoneuroendocrinology. 32(2). 183–191. https://doi.org/10.1016/j.psyneuen.2006.12.004.

Petrowski, K., Conrad, R., 2019. Comparison of Cortisol Stress Response in Patients with Panic Disorder, Cannabis-Induced Panic Disorder, and Healthy Controls. Psychopathology. 52(1). 26–32. https://doi.org/10.1159/000496559.

Prall, S. P., Larson, E. E., Muehlenbein, M. P., 2017. The role of dehydroepiandrosterone on functional innate immune responses to acute stress. Stress and Health. 33(5). 656–664. https://doi.org/10.1002/smi.2752.

Rimmele, U., Seiler, R., Marti, B., Wirtz, P. H., Ehlert, U., Heinrichs, M., 2009. The level of physical activity affects adrenal and cardiovascular reactivity to psychosocial stress. Psychoneuroendocrinology. 34(2). 190–198. https://doi.org/10.1016/j.psyneuen.2008.08.023.

Rimmele, U., Zellweger, B. C., Marti, B., Seiler, R., Mohiyeddini, C., Ehlert, U., Heinrichs, M., 2007. Trained men show lower cortisol, heart rate and psychological responses to psychosocial stress compared with untrained men. Psychoneuroendocrinology. 32(6). 627–635. https://doi.org/10.1016/j.psyneuen.2007.04.005.

Schlotz, W., Hammerfald, K., Ehlert, U., Gaab, J., 2011. Individual differences in the cortisol response to stress in young healthy men: Testing the roles of perceived stress reactivity and threat appraisal using multiphase latent growth curve modeling. Biological Psychology. 87(2). 257–264. https://doi.org/10.1016/j.biopsycho.2011.03.005.

Scholz, U., Marca, R. L., Nater, U. M., Aberle, I., Kliegel, M., 2009. Go no-go performance under psychosocial stress: beneficial effects of implementation intentions. Neurobiology of Learning and Memory. 91(1). 89–92. https://doi.org/10.1016/j.nlm.2008.09.002.

Sep, M. S., van Ast, V. A., Gorter, R., Joëls, M., Geuze, E., 2019. Time-dependent effects of psychosocial stress on the contextualization of neutral memories. Psychoneuroendocrinology. 108. 140–149. https://doi.org/10.1016/j.psyneuen.2019.06.021.

Shalev, I., Lerer, E., Israel, S., Uzefovsky, F., Gritsenko, I., Mankuta, D., Eb Stein, R. P., Kaitz, M., 2009. BDNF Val66Met polymorphism is associated with HPA axis reactivity to psychological stress characterized by genotype and gender interactions. Psychoneuroendocrinology. 34(3). 382–388. https://doi.org/10.1016/j.psyneuen.2008.09.017.

Shiban, Y., Diemer, J., Brandl, S., Zack, R., Mühlberger, A., Wüst, S., 2016. Trier Social Stress Test in vivo and in virtual reality: Dissociation of response domains. International Journal of Psychophysiology. 110. 47–55. https://doi.org/10.1016/j.ijpsycho.2016.10.008.

Shirotsuki, K., Izawa, S., Sugaya, N., Kimura, K., Ogawa, N., Yamada, K. C., Nagano, Y., 2020. Imbalance between salivary cortisol and DHEA responses is associated with social cost and self-perception to social evaluative threat in Japanese healthy young adults. International Journal of Behavioral Medicine. 27(3). 316–324. https://doi.org/10.1007/s12529-019-09835-x.

Shirotsuki, K., Izawa, S., Sugaya, N., Yamada, K. C., Ogawa, N., Ouchi, Y., Nagano, Y., Nomura, S., 2009. Salivary cortisol and DHEA reactivity to psychosocial stress in socially anxious males. International Journal of Psychophysiology. 72(2). 198–203. https://doi.org/10.1016/j.ijpsycho.2008.12.010.

Stephens, M. A. C., Mahon, P. B., McCaul, M. E., Wand, G. S., 2016. Hypothalamic–pituitary–adrenal axis response to acute psychosocial stress: Effects of biological sex and circulating sex hormones. Psychoneuroendocrinology. 66. 47–55. https://doi.org/10.1016/j.psyneuen.2015.12.021.

Strahler, J., Rohleder, N., Wolf, J. M., 2015. Acute psychosocial stress induces differential short-term changes in catecholamine sensitivity of stimulated inflammatory cytokine production. Brain, Behavior, and Immunity. 43. 139–148. https://doi.org/10.1016/j.bbi.2014.07.014.

Suárez-Hitz, K. A., Otto, B., Bidlingmaier, M., Schwizer, W., Fried, M., Ehlert, U., 2012. Altered psychobiological responsiveness in women with irritable bowel syndrome. Psychosomatic Medicine. 74(2). 221–231. https://doi.org/10.1097/PSY.0b013e318244fb82.

Thoma, M. V., Gianferante, D., Hanlin, L., Fiksdal, A., Chen, X., Rohleder, N., 2017. Stronger hypothalamus-pituitary-adrenal axis habituation predicts lesser sensitization of inflammatory response to repeated acute stress exposures in healthy young adults. Brain, Behavior, and Immunity. 61. 228–235. https://doi.org/10.1016/j.bbi.2016.11.030.

Trautmann, S., Muehlhan, M., Kirschbaum, C., Wittchen, H. U., Höfler, M., Stalder, T., Steudte‐Schmiedgen, S., 2018. Biological stress indicators as risk markers for increased alcohol use following traumatic experiences. Addiction Biology. 23(1). 281–290. https://doi.org/10.1111/adb.12487.

van Ast, V. A., Cornelisse, S., Meeter, M., Kindt, M., 2014. Cortisol mediates the effects of stress on the contextual dependency of memories. Psychoneuroendocrinology. 41. 97–110. https://doi.org/10.1016/j.psyneuen.2013.12.007.

van Leeuwen, J. M., Vink, M., Joëls, M., Kahn, R. S., Hermans, E. J., Vinkers, C. H., 2019. Reward-related striatal responses following stress in healthy individuals and patients with bipolar disorder. Biological Psychiatry: Cognitive Neuroscience Neuroimaging. 4(11). 966–974. https://doi.org/10.1016/j.bpsc.2019.06.014.

Villada, C., Espin, L., Hidalgo, V., Rubagotti, S., Sgoifo, A., Salvador, A., 2017. The influence of coping strategies and behavior on the physiological response to social stress in women: the role of age and menstrual cycle phase. Physiology & Behavior. 170. 37–46. https://doi.org/10.1016/j.physbeh.2016.12.011.

Wieck, A., Grassi-Oliveira, R., do Prado, C. H., Rizzo, L. B., de Oliveira, A. S., Kommers-Molina, J., Viola, T. W., Teixeira, A. L., Bauer, M. E., 2013. Differential neuroendocrine and immune responses to acute psychosocial stress in women with type 1 bipolar disorder. Brain, Behavior, and Immunity. 34. 47–55. https://doi.org/10.1016/j.bbi.2013.07.005.

Wilson, M. C., Zilioli, S., Ponzi, D., Henry, A., Kubicki, K., Nickels, N., Maestripieri, D., 2015. Cortisol reactivity to psychosocial stress mediates the relationship between extraversion and unrestricted sociosexuality. Personality and Individual Differences. 86. 427–431. https://doi.org/10.1016/j.paid.2015.07.003.

Wingenfeld, K., Duesenberg, M., Fleischer, J., Roepke, S., Dziobek, I., Otte, C., Wolf, O. T., 2018. Psychosocial stress differentially affects emotional empathy in women with borderline personality disorder and healthy controls. Acta Psychiatrica Scandinavica. 137(3). 206–215. https://doi.org/10.1111/acps.12856.

Wirtz, P. H., Elsenbruch, S., Emini, L., Rüdisüli, K., Groessbauer, S., Ehlert, U., 2007. Perfectionism and the cortisol response to psychosocial stress in men. Psychosomatic Medicine. 69(3). 249–255. https://doi.org/10.1097/PSY.0b013e318042589e.

Wolf, O. T., 2012. Immediate recall influences the effects of pre-encoding stress on emotional episodic long-term memory consolidation in healthy young men. Stress. 15(3). 272–280. https://doi.org/10.3109/10253890.2011.622012.

Wolf, O. T., Schulte, J. M., Drimalla, H., Hamacher-Dang, T. C., Knoch, D., Dziobek, I., 2015. Enhanced emotional empathy after psychosocial stress in young healthy men. Stress. 18(6). 631–637. https://doi.org/10.3109/10253890.2015.1078787.

Zandara, M., Villada, C., Hidalgo, V., Salvador, A., 2018. Assessing the antecedents and consequences of threat appraisal of an acute psychosocial stressor: the role of optimism, displacement behavior, and physiological responses. Stress. 21(4). 304–311.

Zhu, K., Henley, D., Pennell, C., Herbison, C. E., Mountain, J., Lye, S., Walsh, J. P., 2016. Associations between hypothalamic-pituitary-adrenal axis function and peak bone mass at 20years of age in a birth cohort. Bone. 85. 37–44. https://doi.org/10.1016/j.bone.2016.01.016.

Zimmer, P., Buttlar, B., Halbeisen, G., Walther, E., Domes, G., 2019. Virtually stressed? A refined virtual reality adaptation of the Trier Social Stress Test (TSST) induces robust endocrine responses. Psychoneuroendocrinology. 101. 186–192. https://doi.org/0.1016/j.psyneuen.2018.11.010.
